# Committed leadership: a prerequisite for successful implementation of recovery during the workday

**DOI:** 10.1017/S1463423625000027

**Published:** 2025-01-27

**Authors:** Lina Ejlertsson, Annika Brorsson

**Affiliations:** 1 Department of Clinical Sciences, Lund University, Malmö, Sweden; 2 Center for Primary Health Care Research, Region Skåne and Lund University, Malmö, Sweden

**Keywords:** employee health, leadership, primary health care, recovery, wellbeing, workplace intervention

## Abstract

**Aim:**

The aim of this study was to explore the role of managers and employees with an assigned responsibility (i.e. inspirers) when integrating recovery-enhancing activities into everyday work in a primary health care setting.

**Background:**

The possibility of recovery during the workday is essential for employee wellbeing. However, the literature on workplace interventions focusing on recovery is scarce. Especially with regard to the importance of local driving forces, like managers and inspirers.

**Methods:**

Two focus groups and two individual interviews were conducted in this qualitative interview study. In total, ten managers and inspirers from different primary health care centres were interviewed about their experiences of brief recovery interventions at their workplaces. A semi-structured interview guide was used, and the qualitative analysis was conducted by using systematic text condensation.

**Findings:**

From a leadership perspective, two themes with promoting factors for recovery interventions were identified. These were structural promoting factors (including authorisation, communication, and integration) and cultural promoting factors (including attitude, support, and open-mindedness). This knowledge can contribute to future workplace environment development with the focus on recovery during the workday. The results also showed several positive effects of integrated recovery, both on an individual and group level. Hence, this study is a valuable addition to the work recovery research, in terms of understanding the importance of investing in recovery at work.

## Introduction

A robust primary healthcare service with a sufficient supply of primary health care physicians, nurses, and other professionals can offer accessibility, continuity, person- centredness, and coordination. This has been shown to increase quality, reduce costs, and promote equity (Starfield *et al*., 2005). A large-scale mixed methods study from the US used a survey with a sample of patients, primary care clinicians, and health care payers combined with input from experts in the field. The data were qualitatively analysed to develop patient reported items constituting a measure, Person-Centered Primary Care Measure (PCPCM), able to assess high quality primary care. The items confirm the results of Starfield and co-workers (2005), but also include the importance of knowledge regarding the local community and health promotion (Etz *et al*., 2019). In Sweden, primary care comprises a smaller proportion of the entire healthcare system than in most comparable countries. The Organization for Economic Co-operation and Development (OECD) shows that general practitioners (GPs) are 14 % of the physicians in Sweden compared to an average of 20 % in EU (OECD, 2023). In a study, 35 OECD countries were compared using the above mentioned PCPCM measure, which showed the lowest score for Sweden (Zyzanski *et al*., 2021).

Thus, the situation is under great pressure with considerable turnover of staff as well as a high sick leave rate. The Covid-19 pandemic combined with an extensive vaccination programme further sharpened these challenges. A good work environment with healthy employees must be prioritised in order to counteract this external pressure, but also to secure high-quality care for the citizens. A health-promoting workplace is characterised not only by the absence of hazards and risks, but an environment with favourable and advantageous effects for the individual employee (Lindberg and Vingård, 2012).

The manager plays an important role in promoting work-related health (Ljungblad *et al*., 2014). A systematic review aiming at identifying predictors for burnout among health care personnel showed that perceived leadership support had the strongest negative association (Meredith *et al*., 2022). A qualitative study interviewing nurses in German hospital and outpatient care emphasised the importance of organising health promoting interventions. The results mentioned several facilitators for enhanced employee participation, like leadership affirming health promoting behaviour, availability of activities, and participation during worktime (Bleier *et al*., 2023). A focus group study concerning Swedish registered and assistant hospital nurses’ work experience and health from a salutogenic perspective (Antonovsky, 1987) indicated strong correlations between work conditions and perceived health status (Andersson *et al*., 2012). Furthermore, a questionnaire study with mixed Swedish primary health care employees showed that the most important factor for the employees´ wellbeing was the possibility of recovery during the workday (Ejlertsson *et al*., 2018a). The experience of recovery during the workday influenced their wellbeing, irrespective of recovery experienced in other contexts than work.

Recovery can be considered as a process, where the individual restores physical, cognitive, emotional, sensory, and social resources that have been spent when they have been exposed to demands, strains, or other challenges in the workplace. In addition, recovery can occur at work as well as outside of work (Zijlstra and Sonnentag, 2006; Sonnentag and Fritz, 2015). There are basically two types: on the one hand different breaks, rest, and sleep etc., and on the other activities, for example, physical exercise, culture, and social togetherness (eg. Colombo and Cifre Gallego, 2012). Change between tasks that use different resources can also entail recovery. A focus group study with mixed primary health care employees from Sweden, exploring recovery during the workday, identified three important factors that needed to be prioritised in order to promote wellbeing for primary health care staff (Ejlertsson *et al*., 2018b). These factors were variation, companionship, and manageability. With these factors in mind, the researchers conducted recovery interventions in six primary health care centres (PHCCs). The interventions lasted for one year and were integrated into the daily work with activities tailored according to local conditions. A quantitative (questionnaires) and qualitative (focus group interviews) evaluation showed positive effects on the employees’ wellbeing and perceived recovery as well as on the work climate (Ejlertsson *et al*., 2021a; Ejlertsson *et al*., 2021b).

In these previous studies, the researchers were directly involved in the interventions. This is apparently not feasible if the model should be applied on a larger scale, which implies that the processes should be driven by local employees. Furthermore, the previous research concerning the managers role in integrated workplace recovery is scarce. Hence, the role of the local managers, their knowledge, and attitudes towards recovery at work need to be illuminated. Consequently, the aim of this study was to evaluate interventions at PHCCs from these perspectives.

## Method

### Intervention

Earlier studies in the field of recovery during the workday have shown that recovery is the most important factor for employee health (Ejlertsson *et al*., 2018a) and that an intervention with integrated recovery activities can be a successful course of action for enhancing the wellbeing and the recovery experience of employees (Ejlertsson *et al*., 2021a, 2021b). Based on this, smaller intervention studies were planned at eight PHCCs, from urban and suburban environments, in one region in southern Sweden. Each intervention lasted for four weeks (besides one PHCC who choose a period of eight weeks) and was run by employees with a specially assigned responsibility, called inspirers. Participants in the intervention represented all different professions in primary health care, i.e. nurses, physicians, paramedical staff, and administrative staff. Recovery models with two to three recovery-promoting activities were planned according to the existing abilities, needs, and wishes of each of the participating PHCCs. Results from a previous study on recovery during the workday (Ejlertsson *et al*., 2018b) were also considered together with established methods for increasing wellbeing and recovery, along with decreasing work-related stress and fatigue. The activities were either performed individually, together with co-workers, or with the whole employee group. Examples of activities were micro-breaks like deep breathing and mindfulness, lunch break walks, or joint dancing, yoga, and reflection sessions. All employees decided for themselves which activities they wanted to take part in.

### Setting and participants

After the intervention, all inspirers (i.e. the employees who were assigned a responsibility with carrying out the interventions at their respective PHCC) and all managers at the eight PHCCs were asked to participate in a group interview, to share their experiences of the recovery interventions. Participation was voluntary and resulted in six inspirers and two managers taking part in the focus groups, one with inspirers and one with managers. Those who did not have the possibility to participate in the group interviews due to the allotted time were offered an individual interview, which resulted in two additional interviews: one with an inspirer and one with a manager. In total, ten inspirers and managers participated, where all PHCCs were represented either with an inspirer, a manager, or both.

### Data collection

A qualitative method, with individual and focus group interviews, was chosen as appropriate to explore the variety of the participants’ experiences, opinions, and perceptions. The two authors conducted all interviews via a digital communication platform, where one acted as a moderator and the other as an observer. A semi-structured interview guide was used, with questions like, “What was your experience of being an inspirer during the intervention process?”, “What was your role as a manager during the intervention process?”, and “Which effects of the intervention did you notice?” Additionally, the participants were asked to describe promoting and limiting factors related to integrating recovery-promoting activities into everyday work. The interviews lasted between 30 and 80 minutes and were recorded with a digital voice recorder, and then transcribed verbatim by the authors.

### Data analysis

The analysis was inspired by systematic text condensation according to Malterud (2012), and proceeded through the following stages: reading the entire transcripts individually and repeatedly to get an overall impression of the data and identifying preliminary themes; marking text units with similar content and coding for these; condensing and abstracting the essence within each of the meaning units; merging the codes into broader categories and subcategories, comparing them to the original data, then discussing and adjusting them among the two authors until consensus was achieved. All quotations in the results section are identified by a number, denoting which interview it was expressed in.

## Results

Several areas, for the individual employee as well as in the workgroup, were reported as positively affected by the intervention. These were the wellbeing of the employees, the companionship in the work groups, and the recovery awareness among the intervention participants. The managers and inspirers described various effects on the wellbeing of the employees, such as more focus during work tasks and a higher energy level throughout the workday. Furthermore, a stronger feeling of companionship emerged at the workplaces, where the interviewees depicted a better mood among co-workers, increased psychological safety, and strengthened relationships. The awareness concerning recovery and its various influencing factors evolved through talking about – and acting on – recovery during the workday. This led to new insights and knowledge on recovery among the employees and the managers, which was described in the interviews as being eye opening.

- *I believe there is something companionship-building about doing a joint activity, bringing people together, meeting, focusing on the same thing or being silent together for a while. Something that you usually don’t get the opportunity to do, and my experience is that everyone appreciated it.* (2)
- *I also feel that these short little breaks are very important. When you take them, you get a better focus. Just being aware of it, knowing that everyone at the workplace knows it exists and that what you do can have positive effects. To think about it [micro-breaks] contributes to the work environment.* (1)

Another important benefit of integrating recovery into everyday work that was discussed by the interviewees is that it, from several aspects, can lead to better patient care. For example, an increased focus means that the employee can perform better while a more positive mood means improved personal treatment of the patient. Additionally, healthier employees lead to less sick leave and staff turnover which equals patient continuity. The inspirers also described how their expanded knowledge about recovery could be passed on to the patients.

- *One feels very happy afterwards and the afternoon fatigue is not at all to the same extent as it usually is. It is also significantly easier to keep the mood up if you have patients in the afternoon who are very emotionally demanding. This way, you are better equipped to handle it in a good way.* (1)

Furthermore, the analyses identified two themes with promoting factors enabling the intervention processes, from a leadership perspective: structural promoting factors and cultural promoting factors. The themes contained three and two categories respectively, for structural promoting factors it was authorisation, communication, and integration (Table 1), and for cultural promoting factors it was attitude and support (Table 2).

**Table 1.** Overview of structural promoting factors

Categories	Subcategories
Authorisation	Approval Roles
Communication	Information Reminders
Integration	Maintenance Customisation Simplicity

**Table 2.** Overview of cultural promoting factors

Categories	Subcategories
Attitude	Commitment Open-mindedness
Support	Togetherness Encouragement Acceptance

### Structural promoting factors

#### Authorisation

The interviewees explained how it was important that the health-promoting work was sanctioned by the management or the current manager. There needed to be clarity around the framework as well as assigned responsibilities. The two subcategories were approval and roles.

The first subcategory was *approval* from management and/or manager, which was a valuable prerequisite for promoting recovery behaviours and activities in the workplace. Someone needs to make and execute decisions with regard to the recovery-promoting work, as well as establish clear rules on the implementation process. For example, on the time factor, where time to conduct recovery activities needs to be put aside – preferably scheduled – for.

- *When there was direct support from the management, it created more of a team feeling of ‘Now we support this because it’s a good idea’ and it was clearer that those who wanted to participate felt supported, i.e., that there was someone from the management backing them.* (1)

*Roles* was the second subcategory, where the managers and inspirers talked about the distribution of responsibilities. For succeeding with joint recovery activities, it is crucial for several individuals, i.e. an inspiration group, to have a shared responsibility for planning and running the activities. This creates a group dynamic that can better foster the development of the activities and will also guarantee activity continuity even if someone is absent. In addition, the role of the manager was elucidated with a clear responsibility for delegating the tasks. The manager could take on three different roles in the recovery-promoting work: permissive but passive, supportive, or actively participating and leading activities.

- *It is important for the recovery activities to be maintained and that it is not reliant on just one person, but that there are several who can be responsible for it.* (2)

#### Communication

Communication is about letting the conversation about recovery proceed in different forums at the workplace, as an ongoing part of the ordinary work structure. In addition, using different kinds of communication aids to succeed with integrated recovery during the workday. The two subcategories were information and reminders.

*Information* was the first subcategory. The respondents talked about the importance of constant information flow on when, where, and which activities are offered. It was pointed out that this is a task that lies with the manager and inspirers, and can be conveyed both orally and in writing.

- *Regarding information, that there was clear and concise info about where, when, and how, across several different channels, because it is easy to forget when there is no routine.* (1)

The second subcategory was *reminders*, which seems to be a necessity for bringing about recovery as the busy everyday working life proceeds. The inspirers and the managers reported having a responsibility for doing this, but also stressed the importance of word of mouth between co-workers. Additionally, it can be beneficial to use other types of reminders like digital pop-ups, written notes around the workspace, or e-mails.

- *To remind them in the morning meeting – and also during coffee breaks and lunch breaks – to take time for mini-recovery. And to try to get them involved and ask if they’ve taken any mini recovery breaks.* (1)

#### Integration

The interviewees clarified that factors affecting the integration of recovery and making it a natural part of everyday work were important for succeeding with the recovery-promoting work. The three subcategories were maintenance, customisation, and simplicity.

*Maintenance* was the first subcategory, explained as a way to sustain the activities. The recovery activities, both individual and joint, needed to be initiated, planned, led, and maintained by defined persons in order to function well. Furthermore, it was stressed that making the activities an integrated routine should be done step-by-step over a longer period of time.

- *That it is important that you get into a routine or the way of thinking that it is vital that you take time for yourself, in order for you to be able to cope.* (1)

*Customisation* was the second subcategory, which describes the importance of offering different types of recovery activities at work to meet the unique needs of every individual employee, work group composition, and organisation. Also, when planning, it needs to be considered that it is the right activity at the right time and place. The significance of continuous evaluations of the recovery interventions for enabling necessary adjustments along the way was also discussed.

- *I think the activities fulfil different needs. I also think that we are so different as people regarding our needs. Some people always want people around them, while others prefer to take a step away to recover.* (3)

The third was *simplicity*. The managers and inspirers acknowledged that the activities needed to be simple to carry out, in terms of when, where, and how, to fit into everyday work. It was also mentioned that “something is better than nothing”, which illustrates that even short micro-breaks can be valuable for gaining recovery.

- *I think what worked best was activities that were short, close by, and easy to grasp. For example, when I put on some music and stood up. And then people came by and some joined in; we laughed a bit together.* (1)

### Cultural promoting factors

#### Attitude

The interviewees reflected on how the attitude of the manager, members of the inspiration group, and other employees had a great effect on the possibilities of integrating recovery activities at a workplace. The two subcategories were commitment and open-mindedness.

The first subcategory was *commitment*. The managers and inspirers highlighted that if there is an awareness of and a belief in the importance of recovery, as well as a willingness and motivation to be involved in the process, a lot can be won. Furthermore, there is an individual responsibility for each employee in deciding to prioritise recovery during their workday.

- *That we’re all in agreement that ‘This is how we’ll do things in the future’, and that everyone – or at least enough people – sees the value in keeping it up. That’s when you have a basis, I think, for it to be accepted as a regular part of the workday.* (2)

*Open-mindedness* was the second subcategory, which represents the value of certain personality traits of those participating in a workplace intervention. It seems to be successful when there is a positive approach towards – as well as a curiosity in – recovery as a phenomenon. Hence, it was an advantage for participating individuals who saw opportunities instead of obstacles and were willing to try.

- *In the beginning, when I came up with the idea, there was an openness; I experienced that. That there was an openness and a will. ‘Oh yes. This sounds promising. It can be good for our workplace and for our goals.’* (1)

#### Support

The participants across all interviews spoke at length about the importance of support from managers, inspirers, and co-workers for enabling individual and joint recovery activities. The three subcategories were togetherness, encouragement, and acceptance.

*Togetherness* was the first subcategory, where the interviewees illustrated that it makes it easier if you have someone who invites you to join a recovery activity or helps you to pause work for a moment. Moreover, there seems to be a factor of group influence, which means that the recovery experience sometimes can benefit from gathering and performing the activity together with co-workers.

- *I think that the success factor is that you do it together; I think that is important. I think if everyone had been told to do this [mindfulness practice] individually, I don’t think it would have had nearly the same effect.* (2)

The second subcategory was *encouragement*, which was conveyed from managers to inspirers and employees, but also from the inspirers to the other employees. The inspirers described how encouragement helped when trying various activities, and how positive cheers and feedback fostered doing it again. Both the managers and the inspirers talked about themselves as supporters and motivators during the process.

- *I went around and talked to everyone and tried to motivate them as much as possible. I was this support person trying to keep us motivated and keep it alive so it wouldn’t die out.* (4)

The third subcategory about support was *acceptance*. The importance of a permissive climate with an understanding of recovery and its essentiality was discussed. Recovery needs to be an accepted part of everyday work, where there is a basic – and common – understanding about what recovery is and how it can be achieved. Furthermore, everyone can take the time to meet their individual needs for recovery without feeling guilty or stressed.

- *In some way, I feel that we accept each other in a different way, and it is perfectly fine to say that you are having a tough time. And I probably haven’t experienced that much before where you could sit down and say that.*
- *I experienced it to be positive, to be able to say that ‘Yes, but now I feel that I need a micro-break. And that people just acknowledged it, which was a step forward.* (1)

## Discussion

### Summary of main findings

Our results indicate that sustainable local work with recovery during the workday needs a structure with committed leadership, consisting of both managers and inspirers. This requires approval from the manager and a team with shared responsibility. A continuous information flow and evaluations, with a readiness to adjust the activities accordingly, is also important. An open-minded culture, with mutual support and an acceptance of recovery as a part of everyday work, further facilitates recovery behaviours.

The recovery interventions also yielded several beneficial effects, for the individual as well as for the group. These were calm, joy, energy, and focus as well as strengthened companionship and psychological safety. This in turn could make the encounters with patients more efficient and the employees more resilient in emotionally difficult situations. Indirectly this was believed to reduce stress reactions, sick leave, and staff turnover, which could enhance continuity both towards the patients and within the teams. The knowledge regarding recovery gained by the employees could also be passed on to patients.

### Findings in relation to other studies

An important focus of this study is leadership. First, the managers should acknowledge their important role in promoting recovery. They need to know when they should allow, facilitate, or actively participate. The managers in this study apparently believe themselves to have a role as leaders, which addresses the discussion about the relationship between leadership and management. A systematic review indicates that there are attempts to separate these two concepts, but still they are often used interchangeably (Reichenpfader *et al*., 2015). This review also shows that a leader doesn’t need to be a manager. A group of employees with the assigned task and time set aside to lead activities decreases vulnerability and promotes the durability of an intervention. For example, a combination of top-down and bottom-up approaches, where managers as well as employees are committed to the task (Hakanen *et al*., 2017)

The participants in the present study highlighted a permissive climate as a necessity for recovery at work, including accepting each other’s different recovery needs and encouraging each other to take time to engage in recovery-enhancing activities. In a previous study, one-year long recovery interventions for employees with mixed professions at Swedish PHCCs were evaluated quantitatively and qualitatively (Ejlertsson *et al*., 2021b). The results showed that recovery legitimacy, with a permissive attitude from both managers and co-workers, was essential for the individual decision on performing recovery activities at work. In addition, receiving positive feedback when performing recovery activities, increased the likelihood of doing them again. This demonstrates the importance of continuous communication around different aspects of recovery at work.

Important positive effects of the interventions were improved companionship, acceptance, strengthened relationships, and psychological safety. This in turn increased the promoting factor support thus forming a virtuous circle. A systematic qualitative review concerning health care staff from different countries and professions (Okello and Gilson, 2015) suggested that interpersonal workplace trust enhances the intrinsic motivation of healthcare workers, and also stimulates social interaction and co-operation. These complex mechanisms can strengthen the retention of staff as well as the quality of care. The concept of psychological safety applies to the results of this study. Learning is promoted by open-mindedness; when the organisation encourages team members to try new methods, but also to acknowledge errors and make new attempts without fear of criticism or sanctions (Edmondson, 1999). In a recent large study using longitudinal survey data from mixed healthcare workers in the US indicated that perceived psychological safety could decrease the risk for burnout and turnover irrespective of inadequate staffing or lack of other resources (Bahadurzada *et al*., 2024). A meta-analysis developed the theory further and indicated that psychological safety is a more important prerequisite for learning when the organisation is knowledge-intensive, i.e. when complexity prevails, and creativity and sense making are crucial (Sanner and Bunderson, 2015).

In the present study, increased awareness regarding recovery strengthened the motivation to continue the activities, forming another virtuous circle. Moreover, this awareness was also practised towards the patients, meaning that knowledge could be conveyed to the public. This exemplifies the notion of experience-based learning where knowledge gained through personal experience is more durable and easier to pass on (Kolb and Fry, 1975). Practitioners in primary health care often encounter patients with stress-related disorders and simultaneously experience high levels of stress. In one study from the US, 340 graduate nurse students were offered a course where they could learn and practise stress management techniques, which in the next step could be further disseminated to patients. In free-text comments in the course evaluation students reported increased satisfaction and intention to practice the techniques with future patients (Gregg and Twibell, 2016).

According to a literature study by Elangovan and co-authors (2022) nurses’ turnover can depend on in-work factors, i.e. the professional content, or at-work factors, where the relationships and conditions at the workplace or in the organisation are decisive. This study deals mainly with the at-work factors, which are possible to influence. It is shown that excessive staff turnover is harmful in different aspects. Obviously, the relations with the patients are interrupted, with decreased efficiency as a consequence. The interviewees reflected on recovery as a contributing factor for continuity. Studies have demonstrated that excessive turnover of staff also entails weakened teamwork, lost collective memories in the organisation, and finally less momentum in quality improvement. This applies to a qualitative evaluation of a quality improvement programme including approximately 1500 primary health care practices in the US (Baron *et al*., 2020). An explorative qualitative study in Norwegian home care and nursing homes also identified staff turnover as a barrier for implementation processes (Lyng *et al*., 2024). Moreover, a Norwegian registry-based observational study including a little over 4.5 million citizens listed with a regular GP indicates an association between continuity on one hand and decreased use of out-of-hours services, acute hospital admissions, and mortality on the other (Sandvik *et al*., 2022).

### Strengths and weaknesses

Participation in the interventions as well as in this evaluation was voluntary. It can be speculated that this shows another spectrum of gains and challenges than a compulsory intervention would have done. However, we believe that a successful intervention is dependent on shared responsibility, commitment, and continuing adjustment of the activities according to local needs and wishes. In this study, the focus was on the experiences of managers and other leaders. Therefore, the opinions and experiences of the other employees were only indirectly reported. In previous studies, the managers were not included which means that the present study adds important knowledge.

All interviews were performed within weeks or a few months after the intervention, which decreases the risk of the memories being distorted by time. The primary health care is a work context with a constant high time pressure, which required the researchers to being adaptable with regards to when and how the interviews were performed. Also, the recovery interventions were not conducted during the exact same time at the different PHCCs, but over a period of a couple of months. The interventions were brief, and the evaluation was therefore focused on the experiences of the interviewees. Evaluations after more long-term interventions could offer quantitative data on wellbeing, absenteeism, and staff retention. Also, the number of managers and inspirers participating in the interviews was limited even though it can be considered a strength that all eight PHCCs were represented.

The professional backgrounds of the authors – public health and family medicine – mean that the data were interpreted from different frames of reference and that preconceptions could be critically reviewed.

## Conclusion and implications

It is the leader’s responsibility to provide a structure for the recovery intervention, which includes forming a group with a specific assignment to plan and run the activities as well as permission and time for all employees to participate. With this structure in place, the managers and the employees have a shared responsibility to contribute with ideas and suggestions for adjusted or additional activities. A successful implementation of recovery during the workday requires a combined top-down and bottom-up approach. The leadership needs to be committed and supportive, stating that recovery activities are prioritised. It was also shown that a favourable group process involves joint activities, feedback, and mutual encouragement. Furthermore, the participants reported that considerable gains can be achieved with rather modest resources, like an improved subjective well-being, as well as a strengthened companionship and psychological safety in the employee group. The findings from this qualitative study can be applicable in similar contexts, in this case primary health care in comparable countries as well as other health and social care work contexts. With committed leaders and a structured implementation process recovery during the workday can be promoted. This in turn is likely to contribute to a more sustainable working life.
